# Neuroplasticity and Nervous System Recovery: Cellular Mechanisms, Therapeutic Advances, and Future Prospects

**DOI:** 10.3390/brainsci15040400

**Published:** 2025-04-15

**Authors:** Ligia Gabriela Tataranu, Radu Eugen Rizea

**Affiliations:** 1 Department of Neurosurgery, Carol Davila University of Medicine and Pharmacy, 020021 Bucharest, Romania; radu.rizea@umfcd.ro; 2 Department of Neurosurgery, Bagdasar-Arseni Emergency Clinical Hospital, 041915 Bucharest, Romania

**Keywords:** neural networks, oligodendrocytes, synaptic plasticity, neuroinflammation, biomaterials, neuroprosthetics, bioluminescent optogenetics, neural interfaces

## Abstract

Neuroplasticity, the ability of the nervous system to adapt structurally and functionally in response to environmental interactions and injuries, is a cornerstone of recovery in the central (CNS) and peripheral nervous systems (PNS). This review explores the mechanisms underlying neuroplasticity, focusing on the dynamic roles of cellular and molecular processes in recovery from nervous system injuries. Key cellular players, including Schwann cells, oligodendrocytes, and neural stem cells, are highlighted for their contributions to nerve repair, myelination, and regeneration. Advances in therapeutic interventions, such as electrical stimulation, bioluminescent optogenetics, and innovative nerve grafting techniques, are discussed alongside their potential to enhance recovery and functional outcomes. The molecular underpinnings of plasticity, involving synaptic remodeling, homeostatic mechanisms, and activity-dependent regulation of gene expression, are elucidated to illustrate their role in learning, memory, and injury repair. Integrating emerging technologies and therapeutic approaches with a foundational understanding of neuroplasticity offers a pathway toward more effective strategies for restoring nervous system functionality after injury.

## 1. Introduction

Neuroplasticity, the brain’s ability to adapt and reorganize, is key to recovering from stroke, traumatic brain injury (TBI), and neurodegenerative diseases. Once thought to occur only in early life, it is now known to persist throughout adulthood, enabling new therapeutic approaches. Neuroplasticity supports functional recovery by rerouting neural pathways and forming new connections [1]. Treatments like virtual reality (VR), transcranial magnetic stimulation (TMS), and constraint-induced movement therapy (CIMT) aid stroke rehabilitation, while cognitive training and neuromodulation enhance recovery in TBI. In neurodegenerative diseases, interventions such as exercise, brain stimulation, and cognitive training help slow progression and alleviate symptoms. Advancing neuroplasticity-based therapies offers new hope for neurological rehabilitation [2,3,4].

Variations in neuroanatomy, such as differences in cortical thickness, gray matter volume, and synaptic density, significantly influence individual neuroplastic responses. For instance, structural differences in the hippocampus and prefrontal cortex have been associated with varying capacities for learning and memory, impacting how individuals adapt to new experiences or recover from neurological injuries [5,6]. A defining aspect of neuroplasticity is neurogenesis. Research identifies the hippocampus as a key site of plasticity, which is crucial for learning and memory, though the behavioral effects of neuronal changes are still being explored [6,7]. The peripheral nervous system (PNS) connects the body to the central nervous system (CNS), facilitating sensory and motor communication. While CNS and PNS tissues are distinct, their interactions shape nervous system development and function. Specialized oligodendrocyte lineage cells (OLCs) adopt a sensory identity upon contact with peripheral axons, highlighting dynamic nervous system boundaries. Future research aims to clarify the role and maintenance of these unique non-myelinating OLCs [8,9].

Peripheral nerve transfer (PNT) is a clinical approach to restoring limb function following nerve or spinal cord injury. Despite nerve regeneration, full sensory and motor recovery remains challenging due to neuroplastic adaptations. Following sensory deafferentation, cortical reorganization occurs, with previously inactive brain regions integrating inputs from adjacent sensory fields over time [10,11].

Previous studies have extensively documented specific aspects of neuroplasticity, including mechanisms underlying plasticity in the context of stroke recovery, traumatic brain injury, and neurodegenerative diseases [2,3]. However, these studies often focus narrowly on particular diseases or therapeutic interventions, leaving gaps in our integrated understanding of the cellular and molecular foundations that broadly underpin neuroplasticity across both the central and peripheral nervous systems. The present review bridges these gaps by comprehensively synthesizing the roles of key cellular players (such as Schwann cells, oligodendrocytes, and neural stem cells), emerging therapeutic technologies, and molecular pathways of plasticity. The purpose of this review is to provide an integrated synthesis of current knowledge on the cellular, molecular, and therapeutic aspects of neuroplasticity and nervous system recovery, emphasizing translational insights and future directions that may facilitate the development of novel interventions for neurological rehabilitation.

## 2. Materials and Methods

### 2.1. Type of Review

This manuscript is structured as a narrative review designed to synthesize and critically evaluate the existing research literature on neuroplasticity and nervous system recovery.

### 2.2. Literature Search and Selection Criteria

A comprehensive literature search was conducted using the electronic databases of PubMed, Web of Science, Scopus, and Google Scholar, covering publications from 2015 to 2025. Keywords included “neuroplasticity”, “nervous system recovery”, “cellular mechanisms”, “molecular pathways”, “neurorehabilitation”, “Schwann cells”, “oligodendrocytes”, “neural stem cells”, “nerve regeneration”, and related terms. Publications selected included peer-reviewed reviews, original research articles, clinical trials, and pivotal preclinical studies. Additional studies were identified from references cited in selected articles.

### 2.3. Data Collection and Analysis

Relevant information from selected studies was extracted and analyzed to summarize and categorize key findings according to the following thematic areas: cellular mechanisms, molecular pathways, innovative therapeutic strategies, and neurorehabilitation techniques.

## 3. Neuroplasticity and Regenerative Mechanisms in Neural Recovery

Neural adaptability is crucial for recovery after cerebrovascular accidents and traumatic injuries, as it enables the modulation of neural circuits and synaptic connections. This understanding has led to innovations such as deep brain stimulation, non-invasive modulation, neuropharmacological interventions, physical exercise, and cognitive enhancement techniques. These approaches, grounded in cerebral plasticity, are being extensively studied for their potential across various neurological conditions. Following peripheral nerve injury, regenerative processes initiate chromatolysis in the proximal nerve segment and Wallerian degeneration in the distal segment. Schwann cells clear debris, recruit macrophages, and form Büngner bands, guiding axonal regrowth. Regenerative-associated genes (RAGs), particularly c-Jun, play a vital role in reprogramming Schwann cells for myelination and repair, with deficiencies in this pathway leading to ineffective regeneration and neuronal loss [12,13].

The brain’s development involves interconnected processes such as cell proliferation, differentiation, and migration, with glial cells playing a pivotal role in neural circuit formation and maintenance. Microglia contribute to neuronal survival, apoptosis, and synaptic refinement, while neuron–glia interactions regulate cell numbers and network activity. Unlike peripheral tissues, the CNS lacks a conventional lymphatic drainage system, but recent discoveries have identified meningeal lymphatic vessels responsible for immune surveillance and cerebrospinal fluid drainage. These vessels exhibit unique structural adaptations influenced by CNS fluid dynamics. Understanding their role in immune cell trafficking could provide insights into neuroinflammatory conditions and CNS infections, which remain a significant research priority due to the lack of effective diagnostics and treatments for many emerging pathogens [14,15,16].

The complexity of the CNS begins early in development through neurulation, where the ectoderm differentiates into the neural tube, forming the brain and spinal cord. This process is tightly regulated, and disruptions can lead to congenital disorders affecting the CNS and peripheral nervous system [17,18]. Skin homeostasis and epidermal homeostasis also rely on stem cell regulation, particularly in the basal layer of the epidermis and hair follicles. Epidermal stem cells, marked by proteins such as α6 integrin and CD34, contribute to tissue regeneration, while mesenchymal stem cells (MSCs) from Wharton’s jelly (WJMSCs) offer promising applications in regenerative medicine. These cells exhibit multipotency, differentiating into various tissues, including bone, cartilage, and even ectodermal structures like hair follicles, suggesting their potential role in neuroregeneration [19,20].

**Figure 1:**
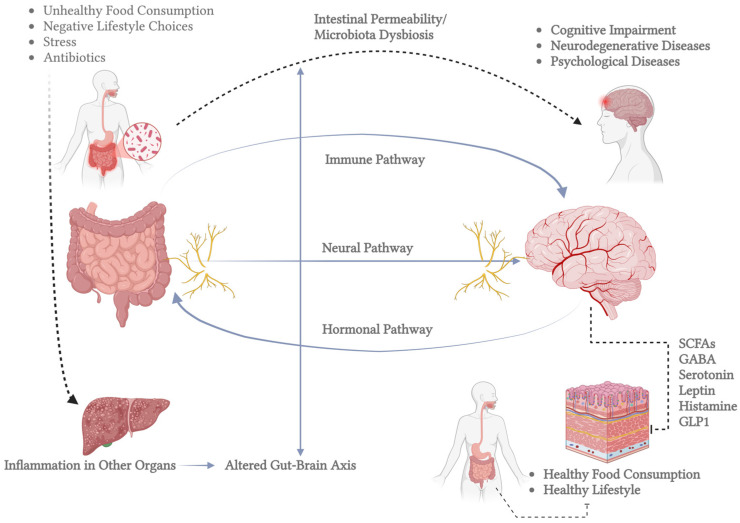
Bidirectional communication between the gut and the brain, known as the gut–brain axis. The left side shows the human gastrointestinal tract and highlights the gut microbiota’s role. The inset magnifies gut bacteria involved in brain-related metabolic processes. Key molecules in gut–brain signaling are listed in the central box, including SCFAs (which regulate inflammation and energy), GABA (which influences mood), serotonin (which affects mood and motility), leptin (which regulates appetite), histamine (which is involved in immune responses and neurotransmission), and GLP1 (which modulates insulin and appetite). The right side depicts the brain, which communicates with the gut through neural, hormonal, and immune pathways. Bidirectional arrows emphasize that signals travel both ways, impacting gut and brain function. This figure highlights the gut–brain axis’s complexity and its role in overall health, pointing to potential therapeutic targets for related disorders (created in BioRender).

The CNS also maintains a network of mononuclear phagocytes, predominantly microglia, originating from embryonic myeloid progenitors and persisting throughout life. Microglia play a crucial role in immune surveillance, synaptic plasticity, and neuronal regeneration by detecting molecular signals of damage and secreting neurotrophic factors [21,22]. Another critical aspect of CNS function is its connection to the gut microbiota, which influences brain activity through neurotransmitter synthesis and metabolic interactions. Microbial metabolites, such as short-chain fatty acids and neurotransmitters like serotonin and dopamine, can modulate brain function through gut–brain signaling pathways. This interaction has been implicated in neurological disorders, including Alzheimer’s, Parkinson’s, and depression, highlighting the microbiota’s role in neuroplasticity and overall brain health [23,24,25]. An important example of the mechanisms underlying neuroplasticity is the gut–brain axis, as illustrated in Figure 1. This axis demonstrates the communication between the gut microbiota and central nervous system function. This figure highlights the pathways through which microbial metabolites and signaling molecules, such as short-chain fatty acids (SCFAs), serotonin, gamma-aminobutyric acid (GABA), and other neuromodulators, influence neural plasticity, inflammatory processes, and overall brain health.

## 4. The Cellular Players in Neural Repair

Schwann cells play an essential role in peripheral nerve regeneration, not only through their ability to myelinate axons but also by actively contributing to the repair process after injury. Their remarkable plasticity allows them to transition into a repair phenotype, facilitating Wallerian degeneration by clearing myelin debris, recruiting macrophages, and forming Büngner bands that guide axonal regrowth. Several molecular pathways tightly regulate this repair process, including the activation of c-Jun, which promotes the Schwann cell repair phenotype and enhances nerve regeneration. The interaction between Schwann cells and immune cells further supports axonal recovery, as cytokines and neurotrophic factors create a microenvironment conducive to neuronal repair. However, in chronic neuropathies such as Charcot–Marie–Tooth disease or diabetic neuropathy, this repair mechanism is often impaired, leading to incomplete regeneration and functional deficits [26,27].

Schwann cells undergo several developmental transitions before assuming their specialized functions. During nerve injury, they exhibit remarkable plasticity, dedifferentiating into repair Schwann cells that clear myelin debris, guide axonal regrowth, and secrete neurotrophic factors essential for regeneration [28]. Additionally, Schwann cells communicate with neurons and surrounding cells through extracellular vesicles (EVs), which transfer bioactive molecules to regulate nerve microenvironments in both health and disease [29].

Following peripheral nerve injuries, Schwann cells facilitate regeneration through Wallerian degeneration, clearing damaged axons and creating a permissive environment for regrowth. Functional recovery is typically rapid in mild injuries (axonotmesis), where connective tissue remains intact. However, regeneration is more complex in severe cases (neurotmesis), where axons and connective sheaths are severed. In combination with nerve guidance conduits and biomaterial scaffolds, Schwann cells are being explored to bridge nerve gaps and enhance repair. Additionally, neurotrophic factors like nerve growth factor (NGF) and p75NTR signaling pathways have been implicated in modulating Schwann cell function, promoting axonal regeneration, and accelerating myelin debris clearance. Advances in Schwann cell transplantation into the central nervous system (CNS) also suggest potential therapeutic applications beyond the PNS, with studies showing their ability to support axonal regrowth and remyelination in spinal cord injuries [30,31,32].

Emerging research focuses on enhancing Schwann cell-based therapies to improve nerve regeneration outcomes. Cellular transplantation approaches using Schwann cells or Schwann cell-like cells derived from induced pluripotent stem cells (iPSCs) are being explored as potential treatments for peripheral nerve injuries. Additionally, strategies such as gene therapy, biomaterial scaffolds, and extracellular vesicle-based signaling modulation aim to optimize Schwann cell function and promote more effective nerve repair. While these approaches show promise in preclinical models, challenges remain in translating them to clinical applications, particularly in ensuring long-term Schwann cell survival and integration into injured nerves. Understanding the molecular mechanisms governing Schwann cell plasticity and their interactions with surrounding tissues will be crucial for developing more effective therapies for peripheral nerve disorders [27,33].

Oligodendrocytes, derived from oligodendrocyte progenitor cells (OPCs), are essential for myelination and repair in the central nervous system (CNS). Their role is particularly significant in diseases like multiple sclerosis (MS), where remyelination often fails despite an inherent capacity for oligodendrocyte production. Studies suggest that new oligodendrocytes are scarce in remyelinated shadow plaques, indicating that existing cells may regenerate myelin rather than new ones forming it. Connexin expression also changes during MS, with reduced Cx32 and Cx47 in oligodendrocytes and increased Cx30 and Cx43 in astrocytes, reflecting altered glial communication. Beyond myelination, oligodendrocytes contribute to neuroprotection and recovery, as seen in ischemic and hemorrhagic strokes. OPCs proliferate and mature to repair white matter damage caused by iron toxicity and cell death. Their diverse functions highlight their importance in maintaining neuronal integrity and responding to CNS injuries and diseases [34,35,36,37].

According to a recent study, oligodendrocytes play a crucial role in central nervous system (CNS) energy metabolism by utilizing fatty acid β-oxidation as an alternative energy source during glucose deprivation. The study demonstrated that oligodendrocytes, unlike astrocytes, can survive prolonged periods of low glucose availability by metabolizing fatty acids within mitochondria and peroxisomes. This process helps maintain ATP levels in axons and prevents conduction blocks, ensuring the preservation of neural function even under metabolic stress. The findings suggest that oligodendrocyte lipid metabolism is a critical energy reserve that supports axonal integrity and function, particularly in white matter tracts where access to extracellular nutrients is limited [1]. Furthermore, disruptions in oligodendrocyte glucose metabolism, such as GLUT1 loss, lead to gradual demyelination, linking imbalanced myelin turnover to neurodegenerative diseases like multiple sclerosis and age-related myelin thinning. The study suggests that oligodendrocytes may support axons through ketogenesis, reinforcing their role in sustaining neuronal function under metabolic stress. These findings highlight their potential as therapeutic targets for CNS disorders involving metabolic dysfunction and demyelination [38].

In addition, oligodendrocytes play a critical role in pain modulation, particularly in neuropathic pain conditions linked to demyelination. Studies indicate that oligodendrocyte loss or dysfunction disrupts sensory processing, contributing to hyperalgesia and allodynia. Additionally, oligodendrocyte precursor cells (OPCs) respond to pain-related demyelination by proliferating, possibly as a compensatory mechanism for myelin repair. However, chronic pain conditions, such as spinal cord injury and chemotherapy-induced neuropathy, are associated with impaired oligodendrocyte differentiation and disrupted myelination, leading to prolonged pain symptoms. Targeting oligodendrocyte survival and myelin repair may offer novel therapeutic strategies for managing chronic pain by restoring normal neural conduction and preventing maladaptive changes in pain pathways [39,40,41].

**Figure 2:**
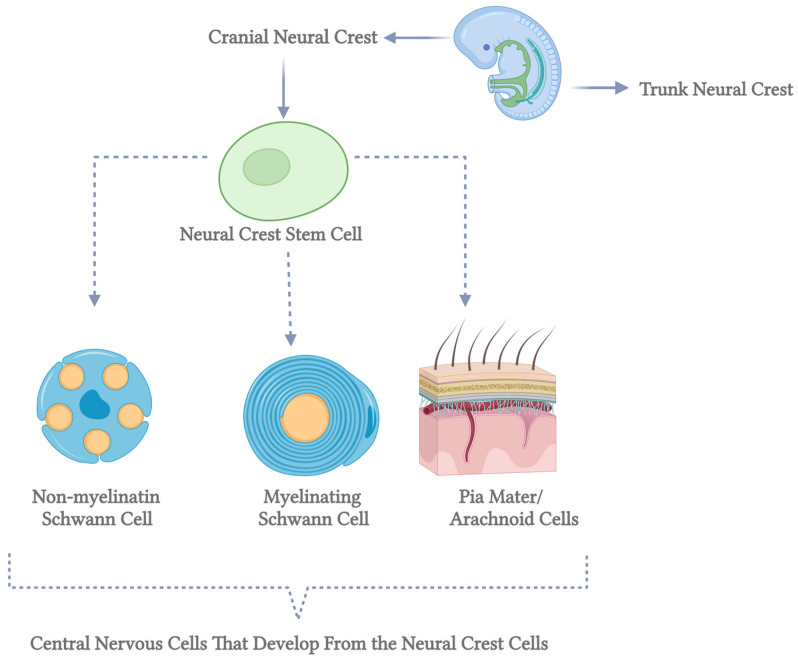
Differentiation of neural crest stem cells into central nervous system cells. This diagram illustrates the developmental pathways of neural crest stem cells into various cell types within the central nervous system. Neural crest stem cells, represented by the green circles on the left, are multipotent stem cells originating from the neural crest during embryonic development. The top pathway shows these stem cells differentiating into non-myelinating Schwann cells, depicted as blue cells with orange nuclei and cytoplasmic extensions. Non-myelinating Schwann cells play a crucial role in maintaining the health of peripheral nerve fibers. The middle pathway illustrates the differentiation into myelinating Schwann cells, shown as blue, characterized by spirally wrapped cells around an axon. These cells are essential for forming the myelin sheath, which insulates nerve fibers and enhances the speed of electrical signal transmission. The bottom pathway demonstrates the development into pia mater and arachnoid cells, represented by a detailed cross-section of the meninges. The pia mater and arachnoid cells are integral components of the meninges, the protective membranes covering the brain and spinal cord. Each pathway highlights the diverse potential of neural crest stem cells to contribute to the formation and function of various essential structures within the central nervous system. This figure underscores the complexity and significance of stem cell differentiation in neurodevelopmental processes (created in BioRender).

Myelin-forming glial cells have distinct roles in axotomized neurons, with Schwann cells in the peripheral nervous system (PNS) promoting neurite extension and remyelination, while oligodendrocytes in the central nervous system (CNS) produce neurite growth inhibitors that hinder axonal regrowth [42]. Oligodendrocytes originate from oligodendrocyte progenitor cells (OPCs), which arise in specific embryonic spinal cord and brain ventral regions before migrating to populate the developing cortex. Over time, early OPC populations are replaced by later waves of cortically derived OPCs through unclear mechanisms. In contrast, Schwann cells derive from neural crest cells that migrate extensively before differentiating into immature Schwann cells. These cells mature into myelinating Schwann cells only when associated with axons larger than 1 μm in diameter, while those interacting with smaller axons form non-myelinating Remak cells. This differentiation process, known as radial sorting, underscores the functional differences between Schwann cells and oligodendrocytes in supporting neuronal repair and regeneration [30] (Figure 2).

Oligodendrocyte progenitor cells (OPCs) play a dual role in both neural regeneration and pathology, with emerging evidence linking them to glioblastoma (GBM) progression and blood–brain barrier (BBB) maintenance. While OPCs are crucial for myelin repair and support neuronal function, studies suggest that factors secreted by GBM cells enhance OPC proliferation, potentially contributing to tumor growth and resistance to chemotherapy. The increased expression of ATP-binding cassette subfamily G member 2 (ABCG2) in GBM cells exposed to OPC-conditioned media suggests a role in drug resistance. At the same time, the upregulation of phosphorylated STAT3 (pSTAT3) further reinforces GBM stemness and radioresistance. Additionally, OPCs have been identified as an integral component of the BBB, where they regulate endothelial tight junction proteins via TGF-β signaling [43,44,45,46]. However, under neurodegenerative conditions like Alzheimer’s disease (AD), OPC function is impaired, leading to compromised myelin formation and axonal degeneration. The inflammatory environment of AD, exacerbated by amyloid-beta (Aβ) accumulation and BBB disruption, further reduces OPC activity, highlighting their critical yet vulnerable role in CNS homeostasis and disease progression [47]. Understanding these interactions may provide new therapeutic targets for both GBM treatment and neurodegenerative disease management.

### 4.1. Neural Stem Cells

Neural stem cells (NSCs) are essential for generating neurons and glial cells in the brain and spinal cord, playing a pivotal role in embryonic development before mainly disappearing in the postnatal central nervous system (CNS). However, in most mammals, NSCs persist in select brain regions, contributing to lifelong neurogenesis, though their presence in the adult human brain remains uncertain. These cells are tightly regulated, as their self-renewal and differentiation disruptions can lead to developmental abnormalities, cognitive impairments, or tumor formation. Notably, signaling pathways governing NSC behavior are also implicated in glioma development [48,49]. In the adult brain, NSCs, known as B1 cells, reside in the ventricular–subventricular zone (V-SVZ), where they share characteristics with astrocytes and originate from radial glia (RG) cells. These B1 cells generate neuroblasts that migrate to the olfactory bulb (OB) and differentiate into inhibitory interneurons, with their regional fate potentially linked to early embryonic forebrain patterning. Although the neuroepithelial-RG-B1 lineage is proposed as the central NSC continuum, it remains unclear whether B1 cells follow this lineage or diverge during development [50].

Hippocampal neural stem/progenitor cell (NSPC) activation and neurogenesis play a key role in recovery after traumatic brain injury (TBI). Following injury, Nestin-expressing NSPCs in the dentate gyrus generate new neurons essential for restoring spatial learning and memory. Apolipoprotein E (ApoE), mainly secreted by astrocytes, regulates neuronal differentiation, and its deficiency shifts NSPCs toward astrogenesis, potentially impairing cognitive recovery [51].

In the spinal cord, ependymal cells lining the central canal serve as a reservoir for neural repair. Although their regenerative capacity in mammals is limited, they exhibit reactive proliferation and neural stem cell properties following spinal cord injury (SCI). Immunofluorescence staining and Western blot analyses have confirmed their differentiation into neurons, astrocytes, and oligodendrocytes, highlighting their potential for CNS regeneration [52].

Unlike the central nervous system (CNS), peripheral nerves retain regenerative potential through the coordinated actions of Schwann cells, macrophages, and neurotrophic factors. However, regeneration is tightly regulated by both growth-promoting and inhibitory molecules, including semaphorins, netrins, and myelin-associated inhibitors (MAIs) such as NOGO and MAG, which bind to receptors like NgR1 and p75, limiting axonal regrowth. Despite these barriers, some spontaneous regeneration occurs, mediated by regenerative signaling pathways that, when disrupted, further hinder axonal recovery. Understanding these mechanisms is essential for developing effective therapies to improve functional restoration after PNS injuries [53,54].

Recent advances in neural stem cell (NSC) research have highlighted their potential for neuroregenerative therapies. Studies have shown that human induced pluripotent stem cell-derived neural stem/progenitor cells (hiPSC-NSCs) exhibit a radial glia-associated signature and maintain long-term stability after transplantation, predominantly differentiating into glial cells without tumor formation. These findings reinforce their viability as a therapeutic alternative to fetal NSCs, particularly for neurodegenerative and demyelinating disorders [55]. Additionally, research has demonstrated that human NSCs can engage in direct intercellular communication through tunneling nanotubes (TNTs), facilitating the transfer of functional mitochondria. This process plays a critical role in rescuing ischemic neurons by preventing apoptosis and restoring bioelectrical activity, offering a novel mechanism of post-ischemic neuroprotection [56]. These discoveries underscore the therapeutic potential of NSCs in neural repair and highlight their multifaceted roles in maintaining neuronal function.

### 4.2. Innovative Approaches in Neural Repair and Rehabilitation

Despite the regenerative capacity of peripheral nerves, complete functional recovery following peripheral nervous system (PNS) injuries remains rare. Factors such as injury severity, location, and treatment timing influence outcomes, with early surgical repair crucial for preserving neuron and target organ integrity. Direct electrical stimulation (ES) has shown promise in enhancing axonal regeneration by activating regeneration-associated genes (RAGs) and promoting neuronal survival. Studies in rodent models have demonstrated improved recovery following ES in crush, transection, and large-gap injuries, highlighting its therapeutic potential [57].

In neurorehabilitation, transcranial magnetic stimulation (TMS) and neuromuscular electrical stimulation (NMES) have been widely studied for improving motor function after stroke. NMES has been shown to relieve muscle spasticity and enhance limb function, while repetitive TMS (rTMS) can promote neuroplasticity and motor recovery. Recent studies suggest that combining NMES with rTMS yields superior results in stroke rehabilitation, significantly improving upper limb function and activities of daily living compared to single therapies [58,59]. Similarly, electrical stimulation therapies have been applied to dysphagia rehabilitation, with NMES proving more effective than traditional therapy for post-stroke swallowing disorders [60]. Additionally, direct electrical stimulation of peripheral nerves, such as the peroneal and tibial nerves, has demonstrated clinical efficacy in improving sensory and motor functions in patients with distal polyneuropathy of the lower extremities. This approach supports the therapeutic versatility of electrical stimulation techniques in neurological rehabilitation beyond post-stroke dysphagia management [61,62].

Furthermore, the clinical efficacy of direct transcutaneous electroneurostimulation of the median nerve has been proven in patients with carpal tunnel syndrome (CTS). Al-Zamil et al. (2023) demonstrated that direct median nerve stimulation effectively facilitated the regression of residual sensory and motor symptoms following carpal tunnel decompression surgery, supporting nerve regeneration and improving functional outcomes [63].

New clinical evidence also demonstrates the potential of direct electrical nerve stimulation techniques in conditions such as transverse myelitis. Nishi et al. (2022) reported significant alleviation of dysesthetic symptoms in patients experiencing spinal nerve dysfunction secondary to transverse myelitis, highlighting an innovative therapeutic avenue for managing chronic sensory disturbances associated with spinal cord pathologies [64]. Additionally, direct peroneal nerve stimulation has shown promise in post-stroke rehabilitation. A randomized controlled trial by Kwong et al. (2018) confirmed that bilateral transcutaneous electrical stimulation of the peroneal nerve improved lower limb motor function and gait performance in patients with chronic stroke, suggesting significant benefits in motor recovery and functional independence following cerebrovascular events [65].

Beyond neurological rehabilitation, exercise and electrical stimulation contribute to muscle regeneration and overall health, particularly in aging and spinal cord injury (SCI) patients. Exercise-induced myokines promote skeletal muscle stem cell activation and fiber regeneration, with extracellular vesicles (EVs) playing a role in systemic adaptation. In SCI, electrical stimulation has been shown to increase muscle mass, improve metabolic function, and enhance mobility. Functional electrical stimulation (FES) cycling studies have reported increased muscle volume and lean leg mass, demonstrating its effectiveness in maintaining musculoskeletal health post-SCI. However, contraindications such as low bone density, cardiovascular instability, and implanted devices must be considered when implementing these therapies [66,67,68].

Targeting specific neuronal populations in the spinal cord after spinal cord injury (SCI) offers a promising approach for rehabilitation by preserving existing connections and promoting controlled neuronal growth. Optogenetics provides a precise, non-invasive method to stimulate spinal cord neurons, overcoming some limitations of electrical stimulation by selectively activating genetically distinct neural subpopulations or glial cells [69].

Bioluminescent optogenetics (BL-OG) further enhances this technique by using luminopsin molecules, combining a luciferase enzyme with channelrhodopsin, enabling light-driven neuronal excitation upon coelenterazine (CTZ) exposure. To validate its specificity, Gomez-Ramirez tested CTZ and its metabolites, demonstrating that neural activity changes were solely due to bioluminescence-triggered opsin activation, confirming BL-OG as a reliable tool for targeted neuromodulation [70,71].

When a segmental nerve gap prevents primary repair, commercially processed allografts and nerve conduits have emerged as potential solutions. Ideally, direct end-to-end nerve repair should occur within three days, as neurotransmitter presence and fascicular alignment decline over time due to Schwann cell proliferation, fibrosis, and angiogenesis. Autografts remain the gold standard for nerve grafting, preserving native architecture and biology, making them preferable for motor and mixed nerves. However, their use is limited by donor site morbidity, nerve deficits, and neuroma formation. In severe injuries requiring extensive reconstruction, rotational or free flaps may be necessary to provide adequate soft tissue coverage before nerve repair [72,73].

For acute electrical burns of the hand, nerve reconstruction follows soft tissue repair using autologous cable nerve grafting, which supports optimal revascularization and regeneration. This approach offers advantages such as reduced invasiveness, minimized graft tension, and placement in a vascularized bed to enhance healing. Proper tunnel spacing prevents ischemia in the graft core, and subsequent tendon reconstruction can be performed without disrupting nerve continuity. Additionally, local factors at the graft site further promote nerve regeneration, improving functional outcomes [74].

Transplanted neural stem/progenitor cells (NSPCs) can serve as neuronal relays, facilitating axonal reconnection by attracting regenerating host axons and extending their own projections into the spinal cord. Studies have demonstrated robust corticospinal tract (CST) regeneration into NSPC grafts and long-distance axonal growth from transplant-derived neurons, forming functional synapses with host neurons [75,76]. Successful neuronal relay formation requires a continuous graft that fully integrates into the lesion site. However, severe injuries often create a hostile environment that impairs NSPC survival and integration. To address this, fibrin matrices enriched with growth factors have been used to support graft retention, differentiation, and connectivity, improving outcomes in large SCI lesions [77].

## 5. Mechanisms of Neural Plasticity

Neural plasticity enables the nervous system to modify synaptic connections in response to experience or injury, allowing for learning, memory formation, and functional recovery. Synaptic strength is regulated through Hebbian plasticity, which involves long-term potentiation (LTP) and long-term depression (LTD), as well as homeostatic scaling to maintain neural stability. Spike-timing-dependent plasticity (STDP) further refines synaptic modifications based on the precise timing of neuronal activity, highlighting the dynamic nature of neural networks [78,79].

Synaptic remodeling shapes neural circuits throughout life, from early postnatal development to adaptive changes in adulthood. Experience-driven processes refine networks, as seen in ocular dominance plasticity, while aging-related declines in synaptic integrity contribute to cognitive deficits. Structural synaptic changes, including alterations in dendritic spines and extracellular matrix (ECM) dynamics, are crucial in maintaining neural function. The dysregulation of these processes is linked to neurodevelopmental and neuropsychiatric disorders, emphasizing the importance of synaptic plasticity in health and disease [80].

Following traumatic brain injury (TBI), synaptic plasticity is disrupted, contributing to posttraumatic amnesia (PTA) and sleep disturbances that impair neurogenesis and memory consolidation. Studies reveal a strong association between sleep–wake cycle disturbances and recovery stages, with interventions targeting sleep hygiene and pharmacological modulation showing potential benefits in restoring cognitive function. Additionally, synaptic maintenance relies on the continuous turnover of molecules at remote synapses, a process supported by single-cell RNA sequencing (scRNA-seq) techniques that identify transcriptional heterogeneity in neural populations, including oligodendrocytes and astrocytes, further refining our understanding of synaptic regulation [81,82,83].

At the molecular level, synaptic architecture depends on cell adhesion molecules (CAMs) and ECM proteins, which regulate synaptic formation and remodeling. N-cadherin and NrCAM mediate neuron–astrocyte interactions, influencing synapse stability and inhibitory synapse function. The cytokine IL-33 has been identified as a key regulator of synaptic plasticity, promoting ECM turnover and microglia-mediated remodeling to maintain neuronal adaptability. ECM digestion has been shown to restore synaptic density and plasticity, reinforcing the role of microglia and oligodendrocytes in shaping the extracellular environment for optimal neural function [84,85,86,87].

### 5.1. Homeostatic Plasticity: Maintaining the Balance

Homeostatic plasticity plays a crucial role in maintaining neuronal stability by counteracting the self-reinforcing nature of Hebbian plasticity. It ensures that neuronal firing rates remain within an optimal range for information processing, preventing excessive or insufficient activity. This regulation occurs both intrinsically, by adjusting neuronal excitability, and synaptically, by modulating synaptic strength in response to activity fluctuations. Such homeostatic mechanisms are essential for stabilizing neural function amidst continuous synaptic modifications during learning and memory formation [88].

Recent studies suggest that homeostatic plasticity can be induced on rapid timescales through the direct modulation of synaptic transmission, independent of overall neuronal activity. Manipulating postsynaptic receptors or presynaptic release machinery has been shown to elicit homeostatic responses within hours, highlighting the role of spontaneous neurotransmission in maintaining synaptic strength. This ability to dynamically balance neural circuits allows the nervous system to remain both flexible for learning and stable for sustained function, preventing extremes of quiescence or hyperactivity that could disrupt cognitive processes [89].

### 5.2. Molecular Mechanisms

Learning and memory rely on synaptic plasticity, which involves the dynamic remodeling of synaptic proteins to strengthen or weaken neural connections. Each neuron contains thousands of synapses, forming intricate protein networks that regulate circuit activity. Plasticity is essential for memory consolidation, requiring both short-term synaptic modifications, such as increased glutamate release and receptor activity, and long-term changes involving gene transcription and protein synthesis. Key signaling pathways, including MAPK and PKA activation of CREB, play a crucial role in encoding long-term memory by promoting synaptic growth and stability [90,91].

Neurotrophic factors such as brain-derived neurotrophic factor (BDNF) are central to plasticity, driving activity-dependent changes in neuronal structure and function. BDNF regulates dendritic plasticity through its receptor TrkB, which is dynamically trafficked to active synapses, facilitating synaptic strengthening. Inflammation also affects neural plasticity, with cytokines like IL-1β disrupting BDNF signaling, impairing memory and learning, particularly in conditions such as stroke and multiple sclerosis. Additionally, chronic pain and depression have been linked to glutamatergic dysfunction, highlighting the interplay between neurotransmitters, inflammation, and neuroplasticity. Understanding these mechanisms may inform therapeutic strategies for cognitive disorders, neuropsychiatric conditions, and neurodegenerative diseases [92,93].

At the behavioral level, habituation—the process of reducing responses to repetitive stimuli—relies on distinct molecular pathways. Short-term habituation results from synaptic depression, while long-term habituation requires gene expression and protein synthesis. Different species utilize varied molecular mechanisms, such as calcium-secretion coupling loss in Aplysia, synaptic transmission reduction in C. elegans, and CamKII phosphorylation in mammals. Despite these differences, the fundamental principle of synaptic adaptation remains critical for sensory processing and learning across species [94].

### 5.3. Epigenetic Modifications Influencing Neural Plasticity

Epigenetic mechanisms dynamically regulate gene expression in the nervous system without altering DNA sequences, influencing both neurodevelopment and plasticity. Traditionally viewed as heritable molecular modifications, epigenetics now encompasses transient transcriptional changes in postmitotic neurons, allowing for adaptive responses to environmental stimuli. These modifications, often mediated by regulatory proteins, shape neural function and structure throughout life, highlighting the brain’s ability to reorganize in response to experience and external factors [95].

Enhancers, promoters, and transcription factors are key in orchestrating gene activity, particularly in neural plasticity and injury-induced remodeling. Enhancers facilitate chromatin looping to activate transcription, contributing to neurogenesis, synaptic reorganization, and responses to cellular stress. Transcription factors drive neuronal adaptation by modulating gene networks involved in dendritic remodeling and axonal regrowth. Despite significant advances in neuroepigenetics, further research is needed to fully understand the role of epigenetic signatures in neural plasticity and their potential therapeutic applications for neurodevelopmental and neurodegenerative disorders [96].

## 6. Strategies for Enhancing CNS Regeneration

Among syndromes of human CNS injury (stroke, trauma, and spinal cord injury), motor recovery after stroke is an area in which neuroplasticity has been extensively studied. Motor deficits are present in the majority of stroke patients, and the degree of motor recovery can significantly influence whether the stroke results in long-term disability. This is a significant issue, with estimates suggesting that 55–75% of stroke survivors still experience functional limitations and reduced quality of life months after the infarct [97].

Studies on motor recovery after stroke illustrate that multiple forms of neuroplasticity can occur simultaneously. Injury to a region of the motor network can result in spontaneous intra-hemispheric changes. In contrast, cognitive recovery after stroke has been less studied and might be more influenced by diaschisis, which is the remote depression of function in non-injured tissue. Restorative and rehabilitation post-stroke therapies induce a range of brain events that are often similar to those arising during spontaneous recovery, such as a return to a normal degree of laterality [98].

Various categories can be considered in the induction of neuroplasticity, including synaptogenesis, neurogenesis, neuroprotection, and a reduction in molecular etiologies of neurodegeneration. Studies have shown that harnessing neuroplasticity by administering molecular compounds often involves crosstalk between these beneficial functions, leading to synergistic effects in restoring neuroplasticity [99,100,101]. However, there are clear limitations to using molecular compounds to treat neurological problems, including the challenge of crossing the blood–brain barrier and ensuring drug retention within the system to reach the desired targets. The modulation of neuronal migratory and guiding proteins, such as doublecortin (DCX), has been repeatedly shown to successfully promote synaptogenesis and neurogenesis when overexpressed in traumatic brain injuries (TBIs) [102,103]. Brain-derived neurotrophic factor (BDNF) is another extensively studied molecule involved in the modulation of neurogenesis [104]. Given that some methods of harnessing neuroplasticity are physical, while others are psychological, future studies should investigate the synergistic effects when administered concomitantly or at different stages of neuronal restoration. It is important to approach data cautiously, as most findings are pre-clinical and may not be clinically suitable. Additionally, some approaches only measure markers of neuroplasticity without assessing the evident physiological and psychological effects, which may create a significant gap in certain pre-clinical and translational studies on neuroplasticity [105].

Calpains are calcium-dependent cysteine proteases that regulate cellular processes through partial protein truncation, including division, migration, and apoptosis. Their involvement in neurodegeneration and cancer has sparked interest in therapeutic inhibition strategies. Despite their discovery in the brain in 1980 alongside their endogenous inhibitor, calpastatin, their precise role in synaptic transmission remains unclear. While studies suggest that calpain contributes to long-term potentiation (LTP) at glutamatergic synapses, definitive mechanisms linking its activation to synaptic changes are still lacking. Calpain-mediated cleavage has been identified in cytoskeletal, membrane, receptor, and presynaptic proteins, highlighting its broad impact on neuronal function [106,107].

The impairment of synaptic plasticity is closely correlated with various pathological conditions, such as cognitive deficits. The epigenetic factor JADE2 is indispensable for maintaining hippocampal synaptic plasticity and cognitive functions. JADE2 expression increases with enhanced neuronal activity both in vitro and in vivo. Knockdown or genetic deletion of Jade2 in hippocampal CA1 results in impaired structural and functional synaptic plasticity, leading to memory impairment. Conversely, the overexpression of JADE2 in CA1 neurons facilitates hippocampal-dependent learning and memory [108].

The induction of hippocampal LTP requires the synaptic activation of postsynaptic NMDA receptors, subsequent postsynaptic Ca^2+^ entry, the activation of several signal transduction systems, and, in the late phases, gene transcription, new protein synthesis, and changes in the single-channel conductance of AMPA glutamate receptors. Recent evidence suggests that glial cells might represent a critical third element of the synapse, in addition to the pre- and post-synaptic neurons, and that immune diffusible mediators, such as pro-inflammatory cytokines, may play a central role in neuronal network function and during the induction of synaptic LTP [109].

Hydrogels are commonly chosen and adapted as support matrices for neural cultures. Various techniques have been employed to add microstructures to hydrogels for different applications. Common approaches include adding microscale particles with anisotropic shapes that can be aligned with physical force and confining the hydrogel physically to create aligned fibrils [110]. Hydrogels hold promise for delivering treatments for nerve injuries and targeting neurons to enhance axon regeneration and synaptogenesis. Drug delivery particles or tubes can extend the release of a drug but typically require a hydrogel to retain the biomaterial within the lesion cavity or to prolong release further. While hydrogels and particles/tubes do not effectively guide the regeneration of white matter tract axons, guidance conduits or fibers can effectively direct regeneration. There is an opportunity to develop drug delivery biomaterial approaches that provide aligned topography, appropriate mechanical characteristics, and specific drug release profiles to treat spinal cord injury (SCI) more successfully [111].

To determine the number of metabolically active cells within the hydrogel, a fluorescent assay based on the ability of viable cells to reduce an indicator dye (resazurin, CellTiter Blue, Promega, G8080, Madison, WI, USA) was used. Resazurin becomes fluorescent (resorufin) in the presence of metabolically active cells. Cell-laden hydrogels were incubated for 3 h with resazurin at a working concentration of 0.2 mg/mL. To assess Schwann cells’ (SCs) metabolic activity within hydrogels, the fluorescence intensity per cell was compared to 2D controls. SCs were plated on a 12-well plate at densities ranging from 10,000 to 300,000 cells per well. After 24 h, cells were incubated for 3 h with resazurin. The linear response of resorufin fluorescence versus the number of cells in 2D controls was determined, and a metabolic rate was calculated by comparing resorufin fluorescence normalized to the cell population within hydrogels with the corresponding fluorescence in 2D controls of the same cellular population. The total number of cells within the hydrogels was determined by cell nuclei staining with bisbenzimide [112].

Nanofiber scaffolds are based on the idea of fabricating biomimetic structures similar to the scale and morphology of the native extracellular matrix (ECM), which is a nanofiber gel network composed of structural proteins, such as collagen and elastin, and non-structural proteins like glycosaminoglycans. Nanofiber scaffolds are characterized by a nanoscale diameter, high surface area/volume ratio, and high porosity with interconnected pores, providing a large surface area for cell attachment and sufficient space for nutrient and waste exchange [113]. In an experiment, a gelatine nanofiber electrospinning membrane (control group) and a gelatine/curcumin nanofiber electrospinning membrane (experimental group) were treated with a corneal trephine with a diameter of 5 mm. The nanofiber membrane used the “membrane-cell suspension-membrane” sandwich model. Each layer’s superposition involved about 6 μL of cell suspension, comprising six layers. First, the two scaffolds were cultured at 37 °C in a 5% CO_2_ incubator for 40 min without a culture medium. Then, they were cultured in high-glucose DMEM with 10% FBS (Hyclone, Logan, UT, USA) and 1% penicillin–streptomycin at 5% CO_2_ and 37 °C. The medium was changed every 3 days, and the culture medium was gently absorbed [114,115].

Bioengineering approaches are transforming neural research and rehabilitation, with magnetic nanoparticles (MNPs) showing potential for modulating microglial activity. However, concerns remain regarding immune system reactivity and suitability for in vivo applications, necessitating further studies with cell-specific targeting strategies. Additionally, findings indicate that PC12 cells absorb significantly more MNPs than primary neurons, questioning their validity as models for neuronal nanoparticle uptake [116].

Neural prosthetics, including brain-machine interfaces (BMIs) and functional electrical stimulation (FES), are advancing spinal cord injury (SCI) treatment by enabling assistive device control and restoring movement. Furthermore, nanotechnology has revolutionized neural interfaces by developing ultrasmall, flexible, and biocompatible materials, such as colloidal quantum dots (QDs). These optoelectronic materials offer enhanced neural modulation capabilities, holding promise for treating neurological disorders and advancing neural interface technology [117,118].

In addition to structural and molecular interventions, restoring cellular energy metabolism represents a promising strategy to support axonal regeneration. Recent research has shown that enhancing mitochondrial function and promoting ATP production in injured neurons significantly improve axonal growth and functional recovery. For example, Han et al. demonstrated that targeting metabolic pathways to restore cellular energetics after spinal cord injury not only promoted axonal regeneration but also improved motor function in vivo [119]. These findings highlight the therapeutic potential of combining metabolic support with traditional neuroregenerative approaches for more comprehensive CNS repair.

## 7. Results

### 7.1. Neuroplasticity and Regenerative Mechanisms in Neural Recovery

Neuroplasticity facilitates nervous system recovery by enabling structural and functional adaptations following injuries. Central to this process are dynamic cellular and molecular mechanisms, including synaptic remodeling, axonal sprouting, neurogenesis, and remyelination [11,12]. After peripheral nerve injury, Schwann cells initiate Wallerian degeneration, clear myelin debris, recruit macrophages, and form Büngner bands to guide axonal regeneration. At the molecular level, regenerative-associated genes (RAGs), particularly c-Jun, are activated, promoting effective Schwann cell-mediated nerve regeneration [11,12,25,26]. In contrast, CNS regeneration is less robust. Still, it relies on mechanisms of intrinsic plasticity such as the modulation of synaptic connections, cortical map reorganization, and neurogenesis, particularly following stroke or traumatic brain injury [9,10,50,51].

### 7.2. The Cellular Players in Neural Repair

Recovery after nervous system injuries significantly depends on specialized glial and neural stem cells. Schwann cells play a pivotal role in peripheral nerve regeneration by adopting a repair phenotype, clearing myelin debris, recruiting immune cells, and secreting neurotrophic factors essential for neuronal survival and axonal regrowth [25,26,27,29,30,31]. In the CNS, oligodendrocytes and oligodendrocyte progenitor cells (OPCs) are critical for remyelination and repair after injuries or demyelinating diseases such as multiple sclerosis. OPCs also participate in neuroprotection and metabolic support for neurons, as well as the modulation of neuropathic pain [33,34,35,36,37,38,39,40,42,43,44,45,46]. Neural stem cells (NSCs) found in neurogenic brain regions contribute significantly to neural regeneration and neurogenesis following traumatic brain injury or spinal cord injury, supporting neuronal replacement and functional recovery [47,48,49,50,51,54,55].

### 7.3. Mechanisms of Neural Plasticity

Neural plasticity involves dynamic alterations in synaptic strength governed by Hebbian plasticity (long-term potentiation and depression), spike-timing-dependent plasticity (STDP), and homeostatic plasticity mechanisms to maintain neuronal network stability [72,73,82,83]. Synaptic remodeling underlies learning, memory, and adaptation to injury, involving structural changes such as dendritic spine formation, synapse turnover, extracellular matrix (ECM) remodeling, and cell adhesion molecule regulation [74,78,79,80,81]. Molecular pathways crucial to neural plasticity include neurotrophic factors such as brain-derived neurotrophic factor (BDNF), inflammatory cytokines, and epigenetic mechanisms regulating gene expression in response to environmental stimuli [84,85,86,87,89,90].

### 7.4. Strategies for Enhancing CNS Regeneration

Several innovative approaches have been developed to promote CNS regeneration and enhance functional recovery post-injury. Electrical stimulation methods, such as transcranial magnetic stimulation (TMS) and neuromuscular electrical stimulation (NMES), have shown promise in improving neuroplasticity and motor recovery after stroke [56,57,58,59]. Bioengineering strategies, including nerve grafting, hydrogels, biomimetic nanofiber scaffolds, and extracellular vesicles, have emerged as promising methods for facilitating nerve regeneration, providing physical guidance, as well as localized delivery of therapeutic agents to injury sites [66,67,68,69,70,71,98,99,100,101,102,103]. Neural stem cell transplantation, often combined with biomaterials or neurotrophic factors, has shown potential for creating neuronal relays to reconnect damaged spinal circuits and enhance recovery from spinal cord injury. Additionally, bioluminescent optogenetics (BL-OG) has been explored as a novel method to selectively activate specific neuronal populations, improving functional outcomes following severe neural injuries [63,64,65,69,70,71].

## 8. Future Directions

Future research in neural regeneration should increasingly consider the role of cellular metabolism and energy restoration in supporting axonal repair. Recent evidence suggests that enhancing mitochondrial function and promoting ATP production within injured neurons can significantly improve axonal outgrowth and functional recovery. These findings highlight the importance of targeting bioenergetic deficits, especially in spinal cord injury, where impaired energy metabolism can hinder the regenerative potential of neurons. Approaches that stabilize mitochondrial dynamics and restore metabolic homeostasis are emerging as valuable therapeutic strategies alongside conventional neuroregenerative interventions [120].

Another promising area involves using extracellular vesicles (EVs) derived from mesenchymal stem cells (MSCs). These nanosized vesicles carry bioactive molecules such as proteins, lipids, and nucleic acids, contributing to neuroprotection, synaptic plasticity, and inflammation resolution. In preclinical models of neurodegenerative diseases, MSC-derived EVs have been shown to reduce neuroinflammation, promote synaptic repair, and improve cognitive outcomes. Their ability to cross the blood–brain barrier and deliver targeted therapeutic cargo positions them as a powerful tool for central nervous system (CNS) repair [120,121].

Furthermore, the development of engineered EVs has expanded the therapeutic landscape. These vesicles can be bioengineered to enhance their targeting specificity, cargo loading, and stability in circulation. Strategies include surface modification to improve homing to neural tissues and the incorporation of therapeutic molecules such as growth factors, microRNAs, or mitochondrial support agents. Combining metabolic enhancement with bioengineered vesicle delivery could create synergistic effects, optimizing the regenerative microenvironment and facilitating more effective CNS repair. As these novel approaches continue to evolve, future studies should focus on refining vesicle production techniques, ensuring reproducibility, and evaluating safety profiles in clinically relevant models [122].

AI and machine learning (ML) are increasingly shaping neuroscience by enhancing our understanding of neural recovery mechanisms. By modeling biological intelligence, AI has the potential to refine rehabilitation strategies, improve patient outcomes, and accelerate discoveries in neuroregenerative medicine. Integrating AI with neuroscience strengthens algorithmic development and offers innovative approaches to neurorehabilitation and cognitive recovery [123]. As research continues to bridge these fields, the potential for developing effective therapies for CNS repair grows, offering new hope for patients with neurological injuries and disorders.

## 9. Conclusions

Neuroplasticity is a fundamental property of the nervous system, enabling structural and functional adaptation in response to injury, disease, or environmental stimuli. This review has provided a comprehensive overview of the cellular and molecular mechanisms underlying neuroplasticity and neural regeneration, particularly emphasizing the roles of Schwann cells, oligodendrocytes, neural stem cells, and immune modulators in creating a supportive microenvironment for repair. Integrating these cellular players with neurotrophic factors and inflammatory signals is critical for facilitating axonal regeneration, remyelination, and synaptic remodeling.

Furthermore, we have outlined various therapeutic strategies to enhance central nervous system (CNS) regeneration, including electrical stimulation, stem cell therapy, tissue engineering approaches, and emerging applications of extracellular vesicles. These strategies offer promising avenues to boost endogenous repair mechanisms, modulate neuroinflammation, and restore neural connectivity. Notably, novel approaches such as bioengineered extracellular vesicles and metabolic enhancement therapies represent a new frontier in neuroregenerative medicine, offering targeted, minimally invasive, and scalable solutions for clinical translation.

Despite substantial progress, challenges remain in translating these findings into effective human therapies, including delivery issues, long-term efficacy, and integration with existing clinical protocols. Future research should focus on multimodal approaches that combine biological, technological, and pharmacological interventions to create personalized and sustainable treatment strategies. By deepening our understanding of neuroplasticity and embracing innovative therapeutic technologies, the field moves closer to realizing effective interventions for a broad range of neurological conditions.
